# Long-term trajectories of reproductive and pituitary-adrenal-thyroid hormones in young males following Omicron BA.2 infection: a two-year prospective cohort study

**DOI:** 10.3389/fendo.2026.1788453

**Published:** 2026-03-31

**Authors:** Fengzhi Li, Yijin Zan, Kun Zhang, Fan Liu, Bingxin Si, Lela Lin, Jing Guo, Qingling Zhang, Dong Wang, Xianrong Xu

**Affiliations:** 1 Department of Respiratory and Critical Care Medicine, Air Force Medical Center, Beijing, China; 2 Department of Respiratory and Critical Care Medicine, The Fifth People’s Hospital of Datong, Datong, China; 3 Department of Healthcare and Diagnostics, Air Force Medical Center, Beijing, China; 4 Vertigo Center, Air Force Medical Center, Beijing, China

**Keywords:** HPG axis, longitudinal study, male reproductive health, omicron variant, SARS-CoV-2, temporal patterns

## Abstract

**Objective:**

To investigate the longitudinal changes and temporal patterns of reproductive, thyroid, and adrenal hormones in young males following SARS-CoV-2 Omicron BA.2 infection, using a 3-month post-infection convalescent reference point.

**Methods:**

A prospective cohort of 71 young male university students with confirmed Omicron BA.2 infection was followed for 24 months. Fasting blood samples were collected at 3 (early convalescent reference), 6, 12, and 24 months post-infection. Serum levels of 15 hormones across the hypothalamic-pituitary-gonadal (HPG), thyroid (HPT), and adrenal (HPA) axes were measured. Linear mixed-effects models were utilized to assess longitudinal changes over time. Persistent symptoms were recorded at each visit.

**Results:**

At the 3-month post-infection convalescent reference point, 8 of 71 participants (11.3%) reported mild persistent symptoms, which decreased to 4.3% by 24 months. Testosterone remained stable throughout the follow-up (all P > 0.05). Relative to the 3-month reference, prolactin declined progressively from an initially elevated mean of 22.55 ng/mL to 14.73 ng/mL at 24 months, gradually normalizing within the clinical reference range (4.04–15.2 ng/mL). Estradiol decreased at all follow-up points (P < 0.0001), while FSH and TSH demonstrated continuous, progressive increases (P < 0.001). TT4 and FT4 showed biphasic fluctuations. Adrenal markers (ACTH, cortisol) generally declined after 6 months (P < 0.0001). Ultimately, most mean hormone values remained within normal clinical reference ranges.

**Conclusion:**

Among young males, testosterone remained robustly stable over two years following Omicron BA.2 infection, while other endocrine markers displayed measurable longitudinal dynamics relative to the 3-month post-infection convalescent reference point. These findings highlight temporal post-infection patterns rather than definitive endocrine injury. While routine extensive endocrine screening may not be necessary for asymptomatic young males, targeted evaluation is recommended for individuals with persistent symptoms.

## 1 Introduction

The Coronavirus Disease 2019 (COVID-19) pandemic has evolved dynamically with the emergence of variants such as Omicron, which exhibits high transmissibility and immune evasion capabilities (1). Although Omicron infections are often associated with milder acute symptoms (2), their potential multisystem temporal impacts warrant further investigation. Clinical studies have indicated sex differences in COVID-19 manifestations, identifying the male reproductive system as a potential area of interest (3).

SARS-CoV-2 interacts with host cells via Angiotensin-Converting Enzyme 2 (ACE2) and Transmembrane Serine Protease 2 (TMPRSS2), both highly expressed in testicular tissue. While early hypotheses raised concerns about severe disruption of the Hypothalamic-Pituitary-Gonadal (HPG) axis (4), current literature is predominantly limited to acute-phase or short-term cross-sectional data. There is a notable paucity of data regarding the long-term temporal patterns (up to two years) of neuroendocrine function following mild Omicron infection, particularly concerning the interplay among the HPG, pituitary-adrenal, and thyroid axes during the extended recovery phase.

To address this gap, we conducted a two-year longitudinal prospective study of young males infected with the Omicron BA.2 variant. Utilizing linear mixed-effects models, we aimed to systematically describe the dynamic trajectories of reproductive, thyroid, and adrenal hormones relative to a 3-month post-infection convalescent reference point. We focus on describing observational temporal patterns after infection rather than drawing definitive causal inferences, acknowledging the inherent limitations of missing pre-infection baselines.

## 2 Materials and methods

### 2.1 Study design and participants

This longitudinal prospective cohort study enrolled 71 male university students (mean age 18.88 ± 0.98 years) admitted to a designated hospital between March 18 and April 28, 2022, with confirmed SARS-CoV-2 Omicron BA.2 infection. All participants had mild-to-moderate acute illness requiring isolation but not intensive care (5). The study was approved by the Ethics Committee of the Air Force Medical Center (Approval No.: 2025-70-YJ01). Written informed consent was obtained from all participants.

### 2.2 Clinical assessments and blood sampling

Follow-up assessments occurred at 3, 6, 12, and 24 months post-infection. The 3-month time point was predefined uniformly as the 3-month post-infection convalescent reference point. At each visit, persistent symptoms potentially associated with long COVID (e.g., fatigue, cognitive impairment, sleep disturbances) were systematically recorded.

Fasting blood sampling was conducted between 8:00 and 9:00 AM. Participants avoided vigorous exercise for 3 days prior and deferred sampling if they had recent acute febrile illnesses. Of the initial 71 participants, all completed the 3-month and 6-month follow-ups. At 12 months, one participant withdrew due to relocation; at 24 months, an additional participant withdrew for personal reasons. Thus, sample sizes were 71 at baseline, 71 at 6 months, 70 at 12 months, and 69 at 24 months.

### 2.3 Biochemical measurements

Serum hormone levels were quantified via chemiluminescence immunoassay (ADVIA Centaur, Siemens Healthcare Diagnostics, UK) with intra-assay coefficients of variation (CV) <5%. Prolactin concentrations were converted from mIU/L to ng/mL using the universally accepted conversion factor (1 ng/mL = 21.2 mIU/L) based on the World Health Organization Third International Standard for Prolactin (IS 84/500), ensuring consistency with standard clinical reference ranges (6). Normal reference ranges and assay limits are detailed in **Supplementary Table 1**.

### 2.4 Statistical analysis

Data were analyzed using R version 4.2.1. Linear mixed-effects models (LMMs) with restricted maximum likelihood (REML) estimation were employed for the 15 hormones, setting time point as a fixed effect and participant as a random intercept to account for within-subject correlation (7). Estimated marginal means (EMMs) with 95% confidence intervals were calculated. Multiple comparisons versus the 3-month post-infection convalescent reference point were adjusted using Tukey’s method.

To calculate the testosterone-to-estradiol (T/E2) ratio in a methodologically rigorous manner, testosterone concentrations were first converted from nmol/L to pmol/L (multiplied by 1000) to ensure uniform units prior to division. Statistical significance was set at P < 0.05.

## 3 Results

### 3.1 Participant characteristics and symptom persistence

At the 3-month post-infection convalescent reference point, 63 of 71 participants (88.7%) reported complete resolution of acute symptoms. Eight participants (11.3%) reported mild persistent symptoms, predominantly fatigue (n=5). Symptom prevalence gradually decreased; by 24 months, only 3 of 69 participants (4.3%) reported non-disabling fatigue. No severe long COVID symptoms were observed (**Supplementary Table 2**).

### 3.2 Baseline hormone levels at the 3-month reference

Descriptive statistics for all 15 hormones at the 3-month reference (n=71) are summarized in **Table 1**. Testosterone levels were within the normal range for all participants. Prolactin levels (after unit conversion) were elevated above the upper limit of normal (15.2 ng/mL) in 62 participants (87.3%), with a mean of 22.55 ng/mL. All other hormones were within their respective normal reference ranges.

**Table 1:** Hormone levels at the 3-month post-infection convalescent reference (n = 71 for all hormones).

Hormone	Mean	95% confidence interval	Standard deviation
Testosterone (nmol/L)	17.25	16.34-18.16	3.83
Prolactin (ng/mL)*	22.55	20.90-24.20	6.92
Estradiol (pmol/L)	135.86	129.70-142.02	25.84
FSH (mIU/mL)	3.58	3.23-3.94	1.49
LH (mIU/mL)	4.82	4.36-5.28	1.9
Progesterone (nmol/L)	3.01	2.69-3.33	1.34
TSH (μIU/mL)	2.29	2.05-2.53	1
TT3 (nmol/L)	2.13	2.07-2.19	0.25
FT3 (pmol/L)	5.8	5.68-5.92	0.5
TT4 (nmol/L)	114.88	109.59-120.17	22.18
FT4 (pmol/L)	12.55	12.23-12.87	1.34
ACTH (pg/mL)	53.14	49.95-56.33	13.41
Cortisol (nmol/L)	475.17	450.25-500.09	96.47
GH (ng/mL)	0.33	0.17-0.49	0.68
PTH (pmol/L)	3.46	3.19-3.73	1.13

Prolactin values converted from mIU/L to ng/mL.

### 3.3 Longitudinal changes in sex hormones

**Figure 1:**
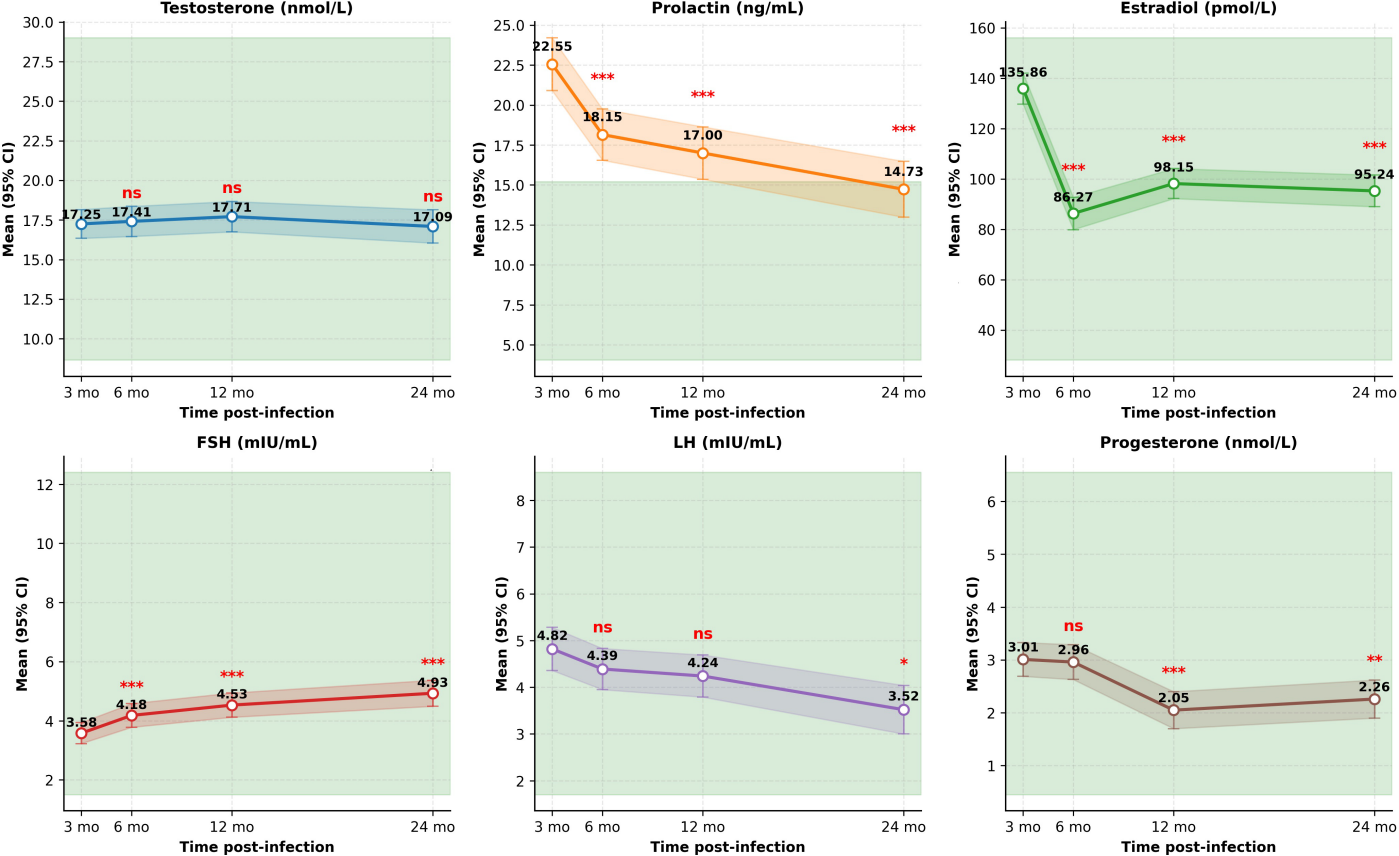
Longitudinal changes in sex hormones relative to the 3-month reference. Points represent estimated marginal means; error bars indicate 95% confidence intervals. Shaded areas denote normal reference ranges. PRL, prolactin; E_2_, estradiol; FSH, follicle-stimulating hormone; LH, luteinizing hormone. Significance symbols: *P < 0.05, **P < 0.01, ***P < 0.001 compared to the 3-month reference (Tukey-adjusted); ns, not significant.

Overall, the HPG axis exhibited stable testosterone levels accompanied by dynamic shifts in gonadotropins and other sex steroids (**Figure 1**, **Table 2**). Specifically, testosterone remained remarkably stable over the two years (all P > 0.05). In contrast, relative to the 3-month post-infection convalescent reference point, prolactin declined progressively (P < 0.0001 at all points), decreasing from the initially elevated 22.55 ng/mL to 14.73 ng/mL at 24 months, thus gradually normalizing within the reference range (4.04–15.2 ng/mL). Estradiol decreased significantly across all follow-up visits (P < 0.0001), while FSH increased progressively (P < 0.0001).

**Table 2:** Estimated marginal means (95% CI) for sex hormones at each time point.

Hormone	Baseline (3 mo)	6 months	12 months	24 months	P vs baseline*
T (nmol/L)	17.25 (16.34–18.16)	17.41 (16.46–18.36)	17.71 (16.75–18.67)	17.09 (16.04–18.14)	0.7535 (6 mo), 0.2791 (12 mo), 0.6187 (24 mo)
PRL (ng/mL)	22.55 (20.90–24.20)	18.15 (16.55–19.75)	17.00 (15.36–18.64)	14.73 (12.98–16.48)	**<0.0001** (all)
E2 (pmol/L)	135.86 (129.70–142.02)	86.27 (79.86–92.68)	98.15 (92.19–104.11)	95.24 (88.97–101.51)	**<0.0001** (all)
FSH (mIU/mL)	3.58 (3.23–3.94)	4.18 (3.78–4.58)	4.53 (4.12–4.94)	4.93 (4.49–5.37)	**<0.0001** (all)
LH (mIU/mL)	4.82 (4.36–5.28)	4.39 (3.95–4.83)	4.24 (3.79–4.69)	3.52 (3.00–4.04)	0.1931 (6 mo), 0.2555 (12 mo), **0.0156** (24 mo)
P (nmol/L)	3.01 (2.69–3.33)	2.96 (2.63–3.29)	2.05 (1.70–2.40)	2.26 (1.90–2.62)	0.4485 (6 mo), **<0.0001** (12 mo), **0.0012** (24 mo)

P values are from linear mixed models comparing each follow-up time point with the 3-month reference, adjusted with Tukey’s method. Bold values indicate statistical significance (P < 0.05). Sample sizes: n=71 at baseline, n=71 at 6 months, n=70 at 12 months, n=69 at 24 months for all hormones.

After unifying measurement units to pmol/L, the T/E_2_ ratio increased from 127 at the 3-month reference to 202 at 6 months, stabilizing around 178–180 at 12 and 24 months, indicating a distinct temporal shift in the testosterone-estradiol balance.

### 3.4 Longitudinal changes in thyroid hormones

**Figure 2:**
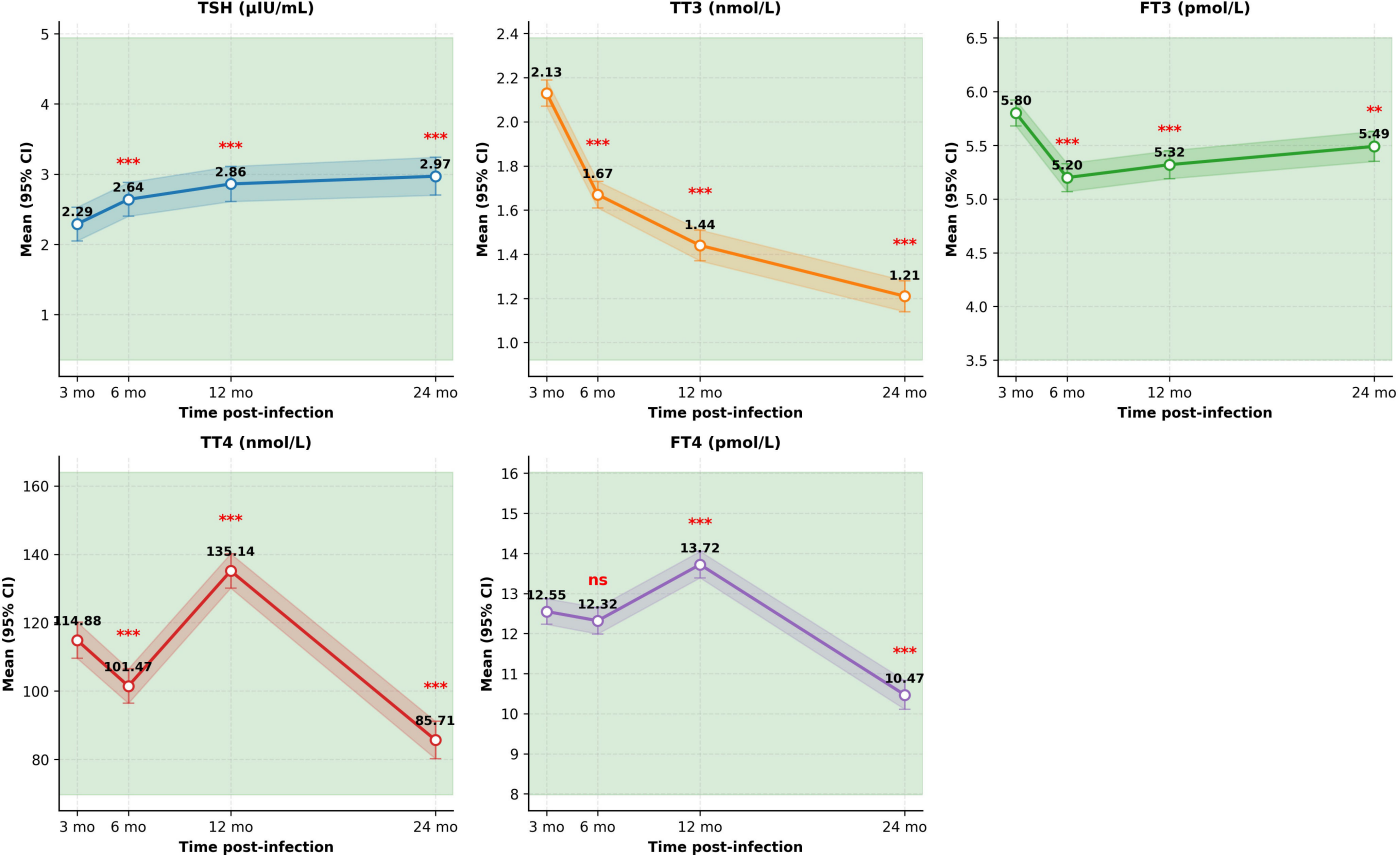
Longitudinal changes in thyroid hormones relative to the 3-month reference. Points represent estimated marginal means; error bars indicate 95% confidence intervals. Shaded areas denote normal reference ranges. TSH, thyroid-stimulating hormone; TT_3_, total triiodothyronine; TT_4_, total thyroxine; FT_3_, free triiodothyronine; FT_4_, free thyroxine. Significance symbols: *P < 0.05, **P < 0.01, ***P < 0.001 compared to the 3-month reference (Tukey-adjusted); ns, not significant.

The HPT axis was characterized by a continuous increase in TSH, alongside biphasic fluctuations in peripheral thyroid hormones (**Table 3**; **Figure 2**). Specifically, TSH levels showed a progressive, sustained elevation from 2.29 μIU/mL at 3 months to 2.97 μIU/mL at 24 months (all P < 0.001). Conversely, TT4 and FT4 demonstrated biphasic temporal patterns, decreasing at 6 months, rebounding at 12 months (P < 0.0001), and declining again at 24 months. Importantly, despite these temporal fluctuations, all mean values of the HPT axis consistently remained within normal clinical reference limits throughout the observation period.

**Table 3:** Estimated marginal means (95% CI) for thyroid hormones at each time point.

Hormone	Baseline (3 mo)	6 months	12 months	24 months	P vs baseline*
TSH (μIU/mL)	2.29 (2.05–2.53)	2.64 (2.40–2.88)	2.86 (2.61–3.11)	2.97 (2.70–3.24)	**0.0003** (6 mo), **0.0004** (12 mo), **0.0005** (24 mo)
TT3 (nmol/L)	2.13 (2.07–2.19)	1.67 (1.61–1.73)	1.44 (1.37–1.51)	1.21 (1.14–1.28)	**<0.0001** (all)
FT3 (pmol/L)	5.80 (5.68–5.92)	5.20 (5.07–5.33)	5.32 (5.19–5.45)	5.49 (5.35–5.63)	**<0.0001** (6, 12 mo), **0.008** (24 mo)
TT4 (nmol/L)	114.88 (109.59–120.17)	101.47 (96.43–106.51)	135.14 (130.11–140.17)	85.71 (80.25–91.17)	**<0.0001** (all)
FT4 (pmol/L)	12.55 (12.23–12.87)	12.32 (11.99–12.65)	13.72 (13.39–14.05)	10.47 (10.11–10.83)	0.0569 (6 mo), **<0.0001** (12, 24 mo)

P values are from linear mixed models comparing each follow-up time point with the 3-month reference, adjusted with Tukey’s method. Bold values indicate statistical significance (P < 0.05). Sample sizes: n=71 at baseline, n=71 at 6 months, n=70 at 12 months, n=69 at 24 months for all hormones.

### 3.5 Longitudinal changes in adrenal hormones

**Figure 3:**
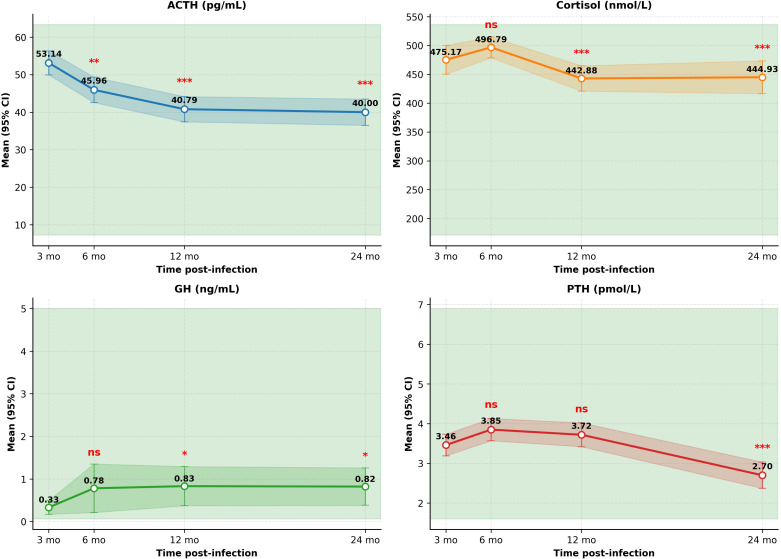
Longitudinal Changes in Adrenal Hormones Relative to the 3-Month Reference. Points represent estimated marginal means; error bars indicate 95% confidence intervals. Shaded areas denote normal reference ranges. ACTH, adrenocorticotropic hormone; PTH, parathyroid hormone. Significance symbols: *P < 0.05, **P < 0.01, ***P < 0.001 compared to the 3-month reference (Tukey-adjusted); ns, not significant.

The HPA axis and related pituitary hormones demonstrated a general pattern of gradual decline from the early convalescent phase (**Table 4**, **Figure 3**). Notably, ACTH and cortisol concentrations decreased progressively after 6 months (P < 0.0001 at 12 and 24 months). Growth hormone (GH) increased slightly at later time points (P < 0.05), while PTH decreased significantly at 24 months (P < 0.0001).

**Table 4:** Estimated marginal means (95% CI) for adrenal hormones at each time point.

Hormone	Baseline (3 mo)	6 months	12 months	24 months	P vs baseline*
ACTH (pg/mL)	53.14 (49.95–56.33)	45.96 (42.57–49.35)	40.79 (37.42–44.16)	40.00 (36.50–43.50)	**0.0033** (6 mo), **< 0.0001** (12, 24 mo)
Cortisol (nmol/L)	475.17 (450.25–500.09)	496.79 (478.71–514.86)	442.88 (420.87–464.89)	444.93 (416.51–473.36)	0.12 (6 mo), **<0.0001** (12, 24 mo)
GH (ng/mL)	0.33 (0.17–0.49)	0.78 (0.21–1.35)	0.83 (0.37–1.29)	0.82 (0.38–1.26)	0.2596 (6 mo), **0.0251** (12 mo), **0.0418** (24 mo)
PTH (pmol/L)	3.46 (3.19–3.73)	3.85 (3.57–4.13)	3.72 (3.42–4.02)	2.70 (2.37–3.03)	0.24 (6 mo), 0.56 (12 mo), **<0.0001** (24 mo)

P values are from linear mixed models comparing each follow-up time point with the 3-month reference, adjusted with Tukey’s method. Bold values indicate statistical significance (P < 0.05). Sample sizes: n=71 at baseline, n=71 at 6 months, n=70 at 12 months, n=69 at 24 months for all hormones.

## 4 Discussion

This two-year prospective cohort study elucidates the longitudinal endocrine temporal patterns across the HPG, HPT, and HPA axes in young males following mild Omicron BA.2 infection. By utilizing a 3-month post-infection convalescent reference point, we observed several key phenomena: (1) testosterone remained robustly stable; (2) initially elevated prolactin progressively declined and normalized; (3) TSH exhibited a continuous progressive increase, whereas peripheral thyroid hormones showed biphasic fluctuations; (4) adrenal markers gradually declined; and (5) the T/E2 ratio indicated a dynamic shift in sex steroid balance.

### 4.1 Reproductive axis changes

In terms of the HPG axis, we observed that testosterone remained remarkably stable, while prolactin declined progressively to normalize within the clinical reference range by 24 months. The initial elevation of prolactin at 3 months may reflect an acute-phase stress response or transient systemic inflammation, which gradually subsided over time. This observation may be explained by altered dopaminergic tone or transient pituitary stress during early recovery (8). The progressive increase in FSH, alongside stable testosterone and changing T/E2 ratios, suggests compensatory mechanisms within the HPG axis. The notable shift in the T/E2 ratio may be associated with modulated aromatase (CYP19A1) activity, which has been reported to be upregulated by SARS-CoV-2 infection (9, 10). Further mechanistic studies are required to validate these hypotheses and confirm whether viral-induced aromatase activity drives these specific sex steroid dynamics.

### 4.2 Thyroid and adrenal axes

For the HPT axis, we clearly observed a progressive increase in TSH over the 24 months, contrasting with the biphasic fluctuations of TT4 and FT4. This pattern suggests a sustained but subclinical central adjustment. Importantly, all mean thyroid values remained within strict normal clinical limits. This aligns with recent large-scale reviews confirming that, despite frequent acute-phase thyroid irregularities, SARS-CoV-2 infection does not typically precipitate major long-term structural thyroid dysfunction (11, 12).

Similarly, the progressive decline in ACTH and cortisol observed from the 3-month post-infection convalescent reference point likely represents a physiological tapering of the HPA axis following the resolution of the acute viral stress response (13). Rather than indicating adrenal injury, this downward trajectory within normal limits reflects expected homeostatic recovery (14).

### 4.3 Clinical implications and long COVID considerations

From a clinical perspective, our findings provide reassuring evidence regarding young males post-Omicron infection. The absolute stability of testosterone and the fact that most hormones remained within reference ranges emphasize a highly practical clinical message: routine, extensive endocrine screening is generally unnecessary for asymptomatic young males recovering from mild COVID-19. However, given that 4.3% of our cohort reported persistent fatigue at 24 months, and considering the known interplay between viral fatigue syndromes, HPA axis tapering, and prolactin dynamics (15), targeted endocrine evaluation remains a prudent recommendation for individuals presenting with sustained or unexplained Long COVID symptoms.

### 4.4 Strengths and limitations

Strengths of this study include its robust two-year longitudinal design with exceptional retention (97.2%), standardized sampling, and rigorous mixed-model statistical analysis. However, limitations must be emphasized: (1) the absence of a pre-infection baseline means the 3-month mark acts only as a post-infection convalescent reference, restricting definitive causal inference regarding the virus; (2) the lack of a non-infected control group prevents the complete exclusion of environmental or age-related temporal variations; and (3) single time-point sampling may not capture the full pulsatile nature of hormones like GH or ACTH.

## 5 Conclusion

In this young male cohort, mild Omicron BA.2 infection was followed by measurable longitudinal temporal patterns in 14 of 15 endocrine markers relative to a 3-month post-infection convalescent reference point, while testosterone levels remained firmly stable. Initially elevated prolactin normalized over two years, and mild, physiological adjustments were noted across the HPT and HPA axes within normal clinical limits. These findings emphasize a generally benign endocrine recovery trajectory, suggesting that while routine widespread screening is unneeded, targeted assessments remain valuable for those with persistent symptoms.

## Data Availability

The raw data supporting the conclusions of this article will be made available by the authors, without undue reservation.
