# Supplementary material for: Long-term trajectories of reproductive and pituitary-adrenal-thyroid hormones in young males following Omicron BA.2 infection: a two-year prospective cohort study
Source: Front Endocrinol (Lausanne). 2026 Mar 31;17:1788453. doi: 10.3389/fendo.2026.1788453 (PMC13076107; doi:10.3389/fendo.2026.1788453)
Supplement: Supplementary file 1 [file Table1.docx]

**Supplementary Material**

**Supplementary Table S1. Assay Characteristics and Reference Ranges for All 15 Hormones**

| **Hormone** | **Method** | **Unit** | **Reference Range** | **LoD** | **Intra-assay CV** |
| --- | --- | --- | --- | --- | --- |
| Testosterone | CLIA | nmol/L | 8.64–29.0 | 0.1 | <5% |
| Prolactin | CLIA | mIU/L (converted to ng/mL) | 85.7–322.2 mIU/L (4.04–15.2 ng/mL) | 3.0 | <5% |
| Estradiol | CLIA | pmol/L | 28–156 | 10 | <5% |
| FSH | CLIA | mIU/mL | 1.5–12.4 | 0.1 | <5% |
| LH | CLIA | mIU/mL | 1.7–8.6 | 0.1 | <5% |
| Progesterone | CLIA | nmol/L | 0.45–6.55 | 0.1 | <5% |
| TSH | CLIA | μIU/mL | 0.35–4.94 | 0.01 | <5% |
| TT3 | CLIA | nmol/L | 0.92–2.38 | 0.1 | <5% |
| FT3 | CLIA | pmol/L | 3.53–7.37 | 0.1 | <5% |
| TT4 | CLIA | nmol/L | 69.71–163.95 | 1.0 | <5% |
| FT4 | CLIA | pmol/L | 7.98–16.02 | 0.1 | <5% |
| ACTH | CLIA | pg/mL | 7.2–63.3 | 1.0 | <5% |
| Cortisol | CLIA | nmol/L | 171–536 (8 AM) | 5.0 | <5% |
| GH | CLIA | ng/mL | 0.06–5.0 | 0.05 | <5% |
| PTH | CLIA | pmol/L | 1.6–6.9 | 0.1 | <5% |

CLIA: chemiluminescence immunoassay; CV: coefficient of variation.

**Supplementary Table S2. Prevalence of Persistent Symptoms at Follow-up Visits**

| Time Point | n | Any Symptom, n (%) | Fatigue, n (%) | Cognitive Impairment, n (%) | Others, n (%) |
| --- | --- | --- | --- | --- | --- |
| 3 months | 71 | 8 (11.3%) | 5 (7.0%) | 3 (4.2%) | 0 (0%) |
| 6 months | 71 | 6 (8.5%) | 4 (5.6%) | 2 (2.8%) | 0 (0%) |
| 12 months | 70 | 5 (7.1%) | 4 (5.7%) | 1 (1.4%) | 0 (0%) |
| 24 months | 69 | 3 (4.3%) | 3 (4.3%) | 0 (0%) | 0 (0%) |

*Note: Data are based on participant self-report at each follow-up visit. "Others" include symptoms such as dyspnea, sleep disturbances, or loss of smell/taste, none of which were reported beyond 3 months.*

**Supplementary Table S3. Sample Sizes for Each Hormone at Each Time Point**

| **Hormone** | **Baseline (3 mo)** | **6 months** | **12 months** | **24 months** |
| --- | --- | --- | --- | --- |
| All 15 hormones | 71 | 71 | 70 | 69 |

Note: Sample sizes reflect participant attendance at each follow-up visit; all hormone measurements were obtained from all attending participants.
